# Global population: from Super-Malthus behavior to Doomsday criticality

**DOI:** 10.1038/s41598-024-60589-3

**Published:** 2024-04-29

**Authors:** Agata Angelika Sojecka, Aleksandra Drozd-Rzoska

**Affiliations:** 1 Department of Marketing, University of Economics in Katowice, ul. 1 Maja 50, 40-257 Katowice, Poland; 2 Institute of High Pressure Physics Polish Academy of Sciences, ul. Sokołowska 29/37, 01-142 Warsaw, Poland

**Keywords:** Global population growth, Super Malthus behavior, Distortions-sensitive analysis, Constrained criticality, Doomsday criticality, Physics of complex systems, Weibull distribution, Historical impacts, Ecology, Evolution, Ecology, Environmental social sciences, Planetary science, Mathematics and computing, Physics

## Abstract

The report discusses global population changes from the Holocene beginning to 2023, via two Super Malthus (SM) scaling equations. SM-1 is the empowered exponential dependence: $P\left(t\right)={P}_{0}exp{\left[\pm \left(t/\tau \right)\right]}^{\beta }$, and SM-2 is the Malthus-type relation with the time-dependent growth rate $r(t)$ or relaxation time τ$(t)=1/r(t)$: $P\left(t\right)={P}_{0}exp\left(r\left(t\right)\times t\right)={P}_{0}exp\left[\tau \left(t\right)/t\right]$. Population data from a few sources were numerically filtered to obtain a 'smooth' dataset, allowing the distortions-sensitive and derivative-based analysis. The test recalling SM-1 equation revealed the essential transition near the year 1970 (population: ~ 3 billion): from the compressed exponential behavior ($\beta >1)$ to the stretched exponential one ($\beta <1$). For SM-2 dependence, linear changes of $\tau \left(T\right)$ during the Industrial Revolutions period, since ~ 1700, led to the constrained critical behavior $P\left(t\right)={P}_{0}exp\left[b{\prime}t/\left({T}_{C}-t\right)\right]$, where ${T}_{C}\approx 2216$ is the extrapolated year of the infinite population. The link to the 'hyperbolic' von Foerster Doomsday equation is shown. Results are discussed in the context of complex systems physics, the Weibull distribution in extreme value theory, and significant historic and prehistoric issues revealed by the distortions-sensitive analysis.

## Introduction

In the twenty-first century, the global population and civilizational globalization become a reality. Technological progress enables the global-scale online exchange of information, increasingly supported by artificial intelligence solutions. Mobile phones and global-scale interpersonal contacts are omnipresent. Industrial activities are often based on global, time-synchronized, supply chains. The imminent implementation of intra-continental ultra-fast railways and inter-continental hypersonic transport will reduce the time required for the global-scale transfer of goods and people to hours. This is the real *Future Shock*^1^ World. However, the spontaneously developing complex and multi-layered global network structure is significantly metastable and threatened by post-imperial paroxysms of some countries, ideological and political madness, and spontaneous waves of migration. But first and foremost, there is a major Climate (Global Warming) & Ecological (Environmental) Crisis, closely related to the Energy Crisis. The response to these fundamental threats and challenges can primarily be the innovations-driven technological transformation towards a sustainable and eco-friendly society, which is matched with supporting 'innovative' socio-economic and political concepts. In the twenty-first century, when the limits of globally available resources and Earth's ecological carrying capacity are widely felt, forecasting trends in global population growth becomes essential^2–9^. Lessons from similar crises in the past may also be important^10–15^.

It is worth returning to the origins of modern considerations on local and global population changes. The seventeenth century saw the birth of the Scientific Method, the cognitive foundation of the Contemporary World^16,17^. Its enormous potential has been proven by the grand works of Isaac Newton, who established the foundations of Modern Science, particularly physics^18–20^. For the Scientific Method, empirical verification is essential. If possible, natural phenomena should be expressed by related scaling equations. Newton puzzled his contemporaries with a common explanation and description of the motion of an apple falling from a tree and the motion of planets and comets in the Sky^18–20^. He also created the mathematical concept of derivatives to formulate appropriate scaling relations^18–20^. In 1798, Robert Malthus declared the inspiration by Isaac Newton's legacy in developing the first scaling equation to describe population changes $P(t)$:^21^

1 $$\frac{dP\left(t\right)}{dt}=rP\left(t\right) \Rightarrow P\left(t\right)={P}_{0}exp\left(rt\right) \Rightarrow lnP\left(t\right)=ln{P}_{0}+rt$$

where $r=const$ is the *Malthusian* or population *growth rate* coefficient, and ${P}_{0}$ is the initial reference population related to the onset time $t=0$.

The world around Malthus was also a significant inspiration. It underwent qualitative changes associated with the 1st Industrial Revolution, Steam Age^22^. Widely implemented innovations could be perceived as ‘magic’ for previous generations people. Examples can be new roads and railways, enabling faster transportation than ever before. Newspapers and developing post services accelerated the dissemination of information. Industrial centers massively produced highly processed goods. Grand expectations for a better life led to migration waves. New industrial cities were overcrowded, shrouded in smog and filth, surrounded by polluted rivers, and rife with poverty and social unrest. But they were also places of hope^22,23^. Does not it remind today’s challenges?

Robert Malthus also noted the crucial role of available resources, originally food, suggesting their linear rise, contrary to the exponential population growth (Eq. 1)^21^. It must lead to resources/food scarcity and a grand crisis with dramatic social and economic consequences. However, the Malthusian crisis has not occured because he did not consider properly the impact of the technological progress, coupled socio-economic changes, and the meaning of international trade. All these are factors that drove the Industrial Revolution^23^. Nevertheless, dictatorial politicians used the 'Malthusian crisis' as a 'false justification’ for aggressive actions against countries^24,25^.

In 1838, Françoise Verhulst^26^ proposed an extension of the Malthus model relation by including explicitly the factor describing available resources:

2 $$\frac{dP\left(t\right)}{dt}=rP\left(t\right)\left[1-\frac{P\left(t\right)}{K}\right] \Rightarrow P\left(t\right)=\frac{K}{1+\left[\left(K-{P}_{0}\right)/{P}_{0}\right]exp\left(-rt\right)}$$

where $K$ is the carrying capacity factor related to the maximum population possible in a given system given the resources available there, materials, food, and ecological, introduced by Pearl and Reed^27,28^.

Originally, Verhulst considered the effect of resources (such as food) via the coefficient $s=1/K$^25^. For $K \to \infty$ or $K\gg P\left(t\right)$ Eq. (2) coincides with Malthus Eq. (1). Notable that the Verhulst equation, is also named the logistic equation or sigmoidal equation.

Two basic cases can be considered for an isolated system with a developing population^27–33^. The first one is related to renewable energy, and the system's carrying capacity remaining constant despite population growth. In the first phase, the population increases following the Malthus pattern (Eq. 1), and subsequently, a transition to the stationary phase with the constant population adapted to available resources amount and quality occurs.

The second case is related to an isolated system with non-renewable resources whose carrying capacity and resources are irreversibly consumed by the growing population. After the first Malthusian growth phase, there is a transition to a short stationary phase. Finally, the decay phase, also named the 'death' phase, with diminishing population occurs.

The rescue from an impending Verhulstian catastrophe may be the third option that can be concluded from Eq. (2). Namely, the evolution or transformation of the population to a new pattern that uses fewer resources and has a limited negative impact on the environment and the ecological carrying capacity. This can mean a change towards the sustainable development pattern for the population. In Verhulst relation (Eq. 2), it can be considered as the modification of the population $P(t)$ itself.

The Malthus and Verhulst relations have found many practical applications and they are modeling tools in microbiology, evolutionary research, and some economic problems^28,29,31,32^. Regarding human populations, one can recall the pattern noted for population changes in apparently non-isolated from the surrounding cities that experienced the development and disappearance guided by a leading and dominated industry: (1) Detroit (IL, USA), former automotive center, (2) Cleveland (Ohio, USA), (3) Bytom (Poland), former coal mining center, (4) Łódź (Poland), former textile industry center^34,35^.

Since the times of Malthus and Verhulst, many models yielding various scaling relations describing global population growth have been developed. They are presented in numerous review reports^36–44^. However, none of them has gained general acceptance or demonstrated the ability to describe a more extended millennial period. A separate problem is the increasing number of parameters in these relations, well beyond the three-parameter optimal reference established in the Verhulst relation, which must result in rising fitting errors^27,28,45,46^.

The second cognitive path of gaining insight into global population changes avoids explicitly formulating a scaling equation and focuses on a targeted statistical analysis of data related to various geographical, socio-economic, and environmental aspects, often starting with local/regional projections in selected areas or for selected social groups. It is frequently broadened by considering migration-related interactions, for instance. Finally, the data are aggregated at the global level. This approach does not have a universal scaling equation to describe local and global population changes^11,46–53^.

However, a modeling framework is required for the statistical analysis at all considered stages. It often recalls models developed in bio-evolutionary research or economics and management, where their effectiveness has been proven. This research path into global population analysis shows some *ex post facto* knowledge features broadly used in social sciences. The significant problem constitutes the uncertainty of error estimations in subsequent stages of the study. For this cognitive path, the maximum possible number of precise data is required for the statistical analysis within a given framework model. However, most of such data is only available globally after 1950. An obvious problem is the final integration, again using *ex-post* adjustments to already known global population values. All these must limit the discussion of past times and burden global population change forecasting. For instance, this type of modeling estimates the global population for the year 2100, ranging from 6.4 to 14.5 billion^49–53^.

Nevertheless, this cognitive path can provide important information about the characteristics accompanying quantitative population changes, including interactions between them, such as education, gender issues, environmental problems, migration, and technological progress. The cohort model approach, the system dynamics (SD) model^49^, the World3 model^51^, and the World4 model^52^ come to mind. Notwithstanding, Bystroff's recent conclusion regarding this path is pessimistic^52^: *‘The field lacks detailed numerical models and objective scrutiny, settling for informal, subjective and descriptive models that are poor predictors of the true numbers that we would like to know.’*

In summary, despite more than two centuries of research, intensified in recent decades, and the practical importance of the problem in an already truly global world, the issue of reliable and fundamentally validated modeling of global population evolution remains a challenge. Notable that problem has been included in the list of the top 125 grand challenges of twenty-first century Science^3^. A typical response to a ‘cognitive crisis’ is the 'return to the roots', which in the given case can mean the re-considering of Malthusian-type or Verhulstian models.

Lehman et al.^46^ recently considered the extended Verhulst (logistic, sigmoidal) concept. They developed the idea of Pearl and Read^27,28^, who suggested that population growth may be associated with a sequence of Verhulst scaling equations, for which a transition to the next carrying capacity-related stage occurs well before reaching the stationary phase. In Ref.^46^, it was related to ecological barriers that can change as the global population grows. It led to the sequence of Verhulst-type scaling equations with 36 pairs of $(r, s)$ coefficients. Very recently, the authors of the given report (AAS, ADR)^31^ considered the linear changes of ${G}_{P}\left(P,t\right)=\left(1/P\right)\left(dP\left(t\right)/dt\right)=dlnP\left(t\right)/dt$, resulting from Eq. (2). It has been proved for empirical global population data for which two linear  ${G}_{P}\left(P\right)$ domains, with the crossover related to the global population $P\approx 3 billion$ occurred in the year $t\approx 1970$ was shown. It led to the conclusion that only a single pair of  $(r,s)$ is allowed but it  does not lead to a representation of global population $P(t)$ data for domains before and after the year $\sim 1970$. However, it does not allow for a reliable portrayal of the global population changes via the Verhulst/logistic Eq. (2). All these can motivate the search for other types of global population models. Notable that the parameter ${G}_{P}$ is a model-independent general characterization of population data referred to as the relative growth rate (RGR) or per capita global population rate coefficient. The above definition is related to the analytic definition introduced in Ref.^31^ Generally, it is implemented in the form appropriate for discrete data with population steps $\Delta P$ for subsequent time-steps $\Delta t$: ${G}_{P}\left(P,t\right)=\left(1/P\left(t\right)\right)\left[\Delta P\left(t\right)/\Delta t\right]$.

In the last decade, numerous reports have indicated that the basic Malthus model should be a re-considered reference for human population growth studies despite its apparent simplicity^54–57^.

This report considers two extended Malthus-type dependences, which we name Super Malthus (SM) scaling relations. The first, named SM-1, is the empowered exponential dependence:

3 $$P\left(t\right)={P}_{0}exp{\left[\pm \left(\frac{t}{\tau }\right)\right]}^{\beta } \Rightarrow lnP\left(t\right)=ln{P}_{0}\pm {\left(t/\tau \right)}^{\beta }$$

where the sign is related to the temporal rise or decay of the considered magnitude; ${P}_{0}$ is the prefactor related to $t=0$ and $\tau$ denotes the relaxation time.

Such dependence is often used in soft matter physics and engineering to portray time-related dependences, which resemble the pattern of global population changes (see the discussion below).

The second, named SM-2, directly recalls Eq. (1) but with the apparent time-dependent growth rate $r(t)$ or relaxation time  $\tau (t)=1/r(t)$:

4 $$P\left(t\right)={P}_{0}exp\left(r\left(t\right)\times t\right)={P}_{0}exp\left(\frac{t}{\tau \left(t\right)}\right) \Rightarrow lnP\left(t\right)=ln{P}_{0}+t/\tau \left(t\right)$$

A significant novelty of the research presented in the given report was the methodology of data preparation and analysis, described below, and recalling concepts developed in studies on complex soft matter systems in physics and material engineering^58–67^. Namely, global population data were taken from a few sources^68–75^ and then numerically filtered to obtain a 'smooth' set for derivation. It enabled the application of the linearized distortion-sensitive analysis, which tests the validity of representing $P(t)$ data via Eqs. (3) and (4) in subsequent time domains. It led to the bottom-up approach, indicating domains of empirical data in which such description is possible and yielding optimal values of basic parameters before the final data analysis of prepared population data via $P(t)$ scaling equations. It is described below, particularly in the Method section.

## Results and discussion

### Remarks on the application of the KWW or Weibull model equation for describing global population changes

The 'empowered exponential' Eq. (3) was first introduced by Rudolf Kohlraush ^76^ to describe the discharge of the Leyden jar, the archetypal capacitor for electric energy storage. In 1970, Graham Williams and David C. Watts^77^ applied this relation to describe dielectric spectra in vitrifying polymers, first in the time domain and after the Fourier transformation in the frequency domain. For decades, the Kohlrausch–Williams–Watts (KWW) relation (Eq. 3) has remained a significant tool in polymer and glass transition physics and material engineering. It is associated with the extreme local deceleration, introducing the non-linear internal time-scale, in supercooled liquid and solid systems before their complete solidification in the amorphous state. The value of the $\beta$ exponent is considered an important metric of the distribution of relaxation times, the canonical feature characterizing complex systems' dynamics. The exponent $\beta =1$ indicates a single relaxation time in a given system and the dominance of the dynamics (temporal changes) by a single process. In such a case, population data dynamics correlates with the basic Malthus Eq. (1). The exponents $\beta \ne 1$ indicate multi-processes dynamics. The stretched exponential case is related to $0<\beta <1$, and ‘slowing’ is coupled to a continuous distribution of relaxation times. For instance, it is observed in supercooled liquids before the amorphous solidification in the glass state^78^ or for near-critical liquids^79^. The compressed exponential behavior is related to $\beta >1$. It indicates the ‘fastened’ dynamics, relatively commonly observed in some material engineering tests^80–82^. Recently, it was also noted in the supercooled state of metallic glass formers^83^. The ‘compressed’ exponential dynamics is a possible general feature for relaxation in the solid glass state^84–86^. Notably, the case $(\beta =2)$ is associated with the normal distribution of relaxation times, describing the complete real-valued random relaxation processes^84,85^.

The KWW-type Eq. (3) mathematically corresponds to the Weibull distribution in extreme values theory, namely the probability distribution function for which the rate of occurrence of events varies with time^87–90^. In such context, the power exponent $\beta$ controls the distribution of the event-related dynamic processes. The time-compressed equation ($\beta >1$) corresponds to the Weibull distribution, where the rate of the event occurrence rises with time. The Weibull model indicates that empowered exponential Eq. (3) can appropriately describe medium and long-time dynamic processes.

Equation (3) also appeared in the nucleation theory introduced by Avrami^91–94^. In such context, the exponent $\beta$ is often called the Avrami constant, and it is theoretically associated with the geometry of the nucleus forming in the chaotic liquid-type surrounding. The Avrami constant is related to the dimensionality $D$ decribing local nucleations: $\beta =D+1$. The surface-related nucleation is related to the dimensionality $D=2$, and then exponent $\beta =3$. The homogeneous nucleation is related to $D=3$ and $\beta =4$.

We emphasize the above general features of dynamics tested via the empowered exponential Eq. (3) in different systems and topics because they offer a supplementary interpretation in analyzing population data, including the global population. For instance, for the global population, clusters of people, from tribes to cities and beyond, can be considered as specifically ordered nuclear centers in disordered surroundings. Following such reasoning, it is significant that modeling studies in the physics of complex systems show that the stretched exponential behavior, with $\beta <1$, is related to slowing-down relaxation and energy dissipation^95^. The exponent $\beta >1$ for accelerated dynamics, above the reference pattern for $\beta =1$, and the system's internal energy amplification/creation. On the other hand, compressed relaxation can reflect the domination of fast local processes, and stretched relaxation can be the indicator of the dominant impact of large-scale processes^96^.

Regarding the practical implementation of (SM-1) Eq. (3) for describing population data, the following dependence can be first considered:

5 $$P\left(t\right)={P}_{0}exp{\left[\pm \left(\frac{t}{\tau }\right)\right]}^{\beta } \Rightarrow lnP\left(t\right)=ln{p}_{0}\pm \frac{{t}^{\beta }}{{\tau }^{\beta }} \Rightarrow \frac{dlnP\left(t\right)}{dt}=\frac{dP\left(t\right)/P\left(t\right)}{dt}=\frac{\beta }{{\tau }^{\beta }}{t}^{\beta -1}=h\left(t\right)$$

In the last part of Eq. (5), notable is the coincidence with the hazard function $h(t)$ in the Weibull probability analysis^88–90^, showing how likely something is to fail given that it has survived so far. Notable is the link with the relative growth rate (RGR) per capita, one of the primary characterizations of population growth:

6 $${G}_{P}\left(t\right)=\frac{1}{P\left(t\right)}\frac{\Delta P\left(t\right)}{\Delta t} \Rightarrow \Delta t\to 0 \Rightarrow \frac{1}{P\left(t\right)}\frac{dP\left(t\right)}{dt}=\frac{dP\left(t\right)/P\left(t\right)}{dt}=\frac{dlnP\left(t\right)}{dt}$$

So far, ${G}_{P}\left(t\right)$ has been used only via the left side of Eq. (6) ^51^, i.e., assuming the discrete representation of empirical data. Numerical filtering of population data can yield a 'smooth' set of data for which the derivative-based analysis is possible, as in the right-hand part of Eq. (6). One can also consider the linearized representation of Eq. (5):

7 $$y\left(t\right)={log}_{10}\left[\frac{dlnP\left(t\right)}{dt}\right]={log}_{10}{G}_{P}\left(t\right)={log}_{10}\left(\frac{\beta }{{\tau }^{\beta }}\right)+\left(\beta -1\right){log}_{10}t=A+B\times x$$

where $t$ is related to the reference onset time ${t}_{0}$; $A={log}_{10}\left(\beta /{\tau }^{\beta }\right)=const$ and $B=\left(\beta -1\right)=const$.

Following Eq. (7), for the plot of transformed empirical population data defined by Eq. (7), namely $y\left(t\right)={log}_{10}{G}_{P}\left(t\right)$ vs. ${log}_{10}t$, emerging linear domains indicate regions where the application of SM-1 Eq. (3) is validated. The following linear regression fit in these linear domains determines optimal values of $A$ and $B$ coefficients, and consequently the power exponent $\beta$ and the relaxation time $\tau$, or the rate coefficient $r=1/\tau$. Such a procedure also provides unambiguous estimates of parameter-related errors, which is often a puzzling issue in population dynamics studies. The final fitting of $P(t)$ data by SM-1 Eq. (3) is then reduced solely to the prefactor ${P}_{0}$.

Regarding the time $t$, it is considered since 12,000 BC. It is ca. 4000 years after the end of the last glaciation. One can treat it as the Holocene 'dawn': the global temperature rose by 4 °C from glaciation termini (16,000 BC), and the ice sheets were retreating all over the planet, freeing up new areas for human expansion^97^. In the case of local anomalies, Eq. (3) is considered from the onset of the local phenomenon.

It is worth noting that for $\beta =1$ in Eq. (3), the relaxation time $ln2\times \tau$ is for the time required for 50% decay or rise of the population. For $\beta \ne 1$ the parameter ${\tau }^{\beta }$ should be considered.

SM-2 Eq. (4) contains time-dependent relaxation time  $\tau \left(t\right)$. It cannot be directly applied to portraying $P\left(t\right)$ data, because it requires the knowledge of $\tau \left(t\right)$ changes pattern in prior. However, one can determine $\tau \left(t\right)$ changes in subsequent time steps via the following dependence, directly resulting from Eq. (4):

8 $$P\left(t\right)={P}_{0}exp\left(\frac{t}{\tau \left(t\right)}\right)={P}_{0}exp\left(r\left(t\right)\times t\right) \Rightarrow r\left(T\right)=1/\tau \left(T\right)=\left(1/t\right)ln\left(P\left(t\right)/{P}_{0}\right)$$

It can reveals population changes in subsequent time domains in changing population.

### The analysis of empirical global population data

**Figure 1:**
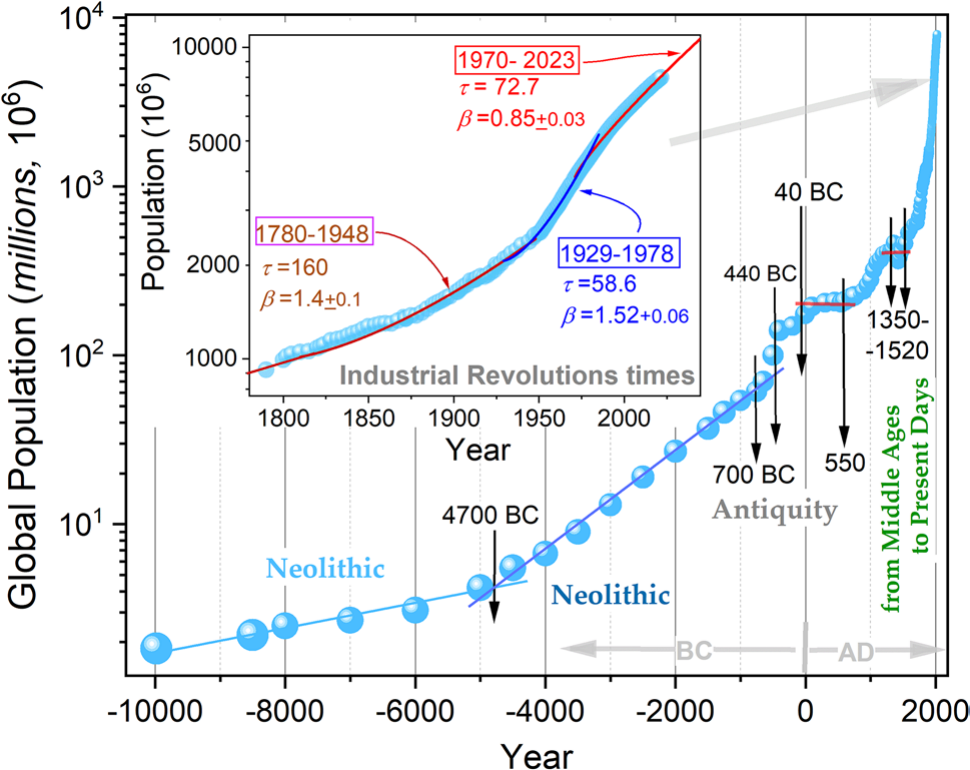
The ‘classic’ plot of the world population growth from the Holocene onset till 2023, based on data described in the Methods Section. Vertical arrows indicate characteristic dates appearing in the plot. Straight lines indicate the basic Malthus behavior (Eq. 1). The inset is focused on the Industrial Revolutions times. It also includes parameterizations via the empowered exponential Super Malthus (SM-1) Eq. (3), as described in the analysis related to Fig. 2.

Figure 1 presents global population changes from the beginning of the Holocene^97^ (10,000 BC) to 2023, based on data prepared as described in the Methods section. The size of the circles for subsequent population data shows a significant increase in uncertainty on shifting away from modern times. The inset focuses on the Industrial Revolutions^23^ period.

For the applied semi-logarithmic scale, the non-linear behavior indicates the behavior beyond the basic Malthus pattern (Eq. 1). The latter seems to appear in prehistoric times, splitting into two domains: (1) from 10,000 BC to 4800 BC (± 500), with the growth factor ${r}_{1}\approx 1.11\times {10}^{-4} ({year}^{-1})$, relaxation time ${\tau }_{1}\approx 8900 (year)$, and (2) from ~ 4800 BC till ~ 800 BC, with ${r}_{2}\approx 6.65\times {10}^{-4} ({year}^{-1})$, i.e., the relaxation time ${\tau }_{2}\approx 1500 (year)$. It is related to the Malthus Eq. (1), or Super-Malthus SM-1 Eq. (3) with the exponent $\beta =1$.

The crossover between domains (1) and (2) coincides with the transition from the early Neolithic to the late Neolithic epoch^98^. In subsequent centuries, the population grew well above these patterns, but from ~ 50–30 BC till ~ 500 AD, an exceptional global population plateau in the global population is visible. It is marked by the red horizontal line in Fig. 1. This period correlates with Roman Empire times, with population of nearly 40 million and probably even 70 million at its peak. At that time, the global population is estimated to 190–200 million^99,100^. The rise, fall, and grand achievement of the Roman Empire is a topic of continuous interest, summarized in number of excellent monographs^99–104^. For this work's authors, however, there is an issue that requires special emphasis due to its possible impact on the global population. In the Roman Empire, enslaved people were exploited in an unprecedented way and scale. For instance, in particular important for the Empire economy Rio Tinto silver mines, tens of thousands of enslaved people could survive only from 6 months to 2 years^102,105^. The weakening of the Empire and the strengthening of its 'barbarian' neighbors limited the 'supplies’ of enslaved people, which could accelerate the imperial economy collapse. For the authors (AAS, ADR), such cruel mass treatment of people as a ‘raw/energetic material’ in Roman Empire period yields an anomaly in global population trends. For millennia slavery was a significant element for social structures and economy. However, the Roman Empire introduced a scale of exploitation that could affect the global population.

After the collapse of the Roman Empire (500 AD) a permanent and accelerating non-Malthusian increase in the global population began, which has continued to the present days. Only the *Black Death* pandemic devastating Asia and Europe's population^106^, left a strong mark.

The inset in Fig. 1 focuses on the Industrial Revolutions period, the new epoch that reshaped the World and Civilization^22,23^. The inset covers the period from 1800 (1 billion population) to 2023 (over 8 billion population).

**Figure 2:**
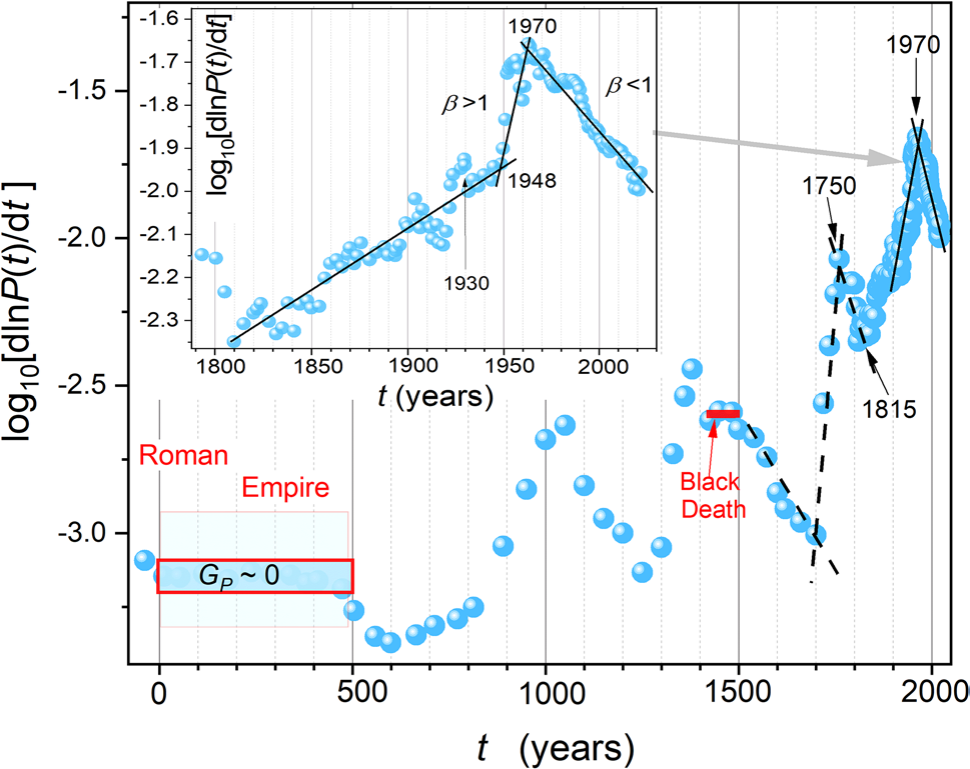
The derivative-based and distortions-sensitive analysis of global population data is shown in Fig. 1, from the onset of the Roman Empire till 2023. The analysis is related to Eq. (7) and indicates domains of the validated portrayal using the empowered exponential SM-1 Eq. (3) and data transformation by Eq. (7). The linear behavior indicates regions where Eq. (3) can be used. Related values of the power exponent are given in the plot. The inset focuses on the Industrial Revolutions period. Note the link to the per capita relative growth rate ${G}_{P}$, defined and commented in the “Introduction”.

Figure 2 presents the distortions-sensitive analysis associated with the validated application of the empowered exponential SM-1 dependence (Eq. 3), emerging as linear domains. The plot is related to ${log}_{10}{G}_{p}\left(t\right)$ evolution in subsequent years, following Eq. (7). ${G}_{P}$ denotes the relative growth rate (RGR) or per capita global population rate coefficient, as above. The plot extends Roman Empire times till today, i.e., it covers 2 millennia. Figure S1 in Supplementary Information broadens the view to the Holocene onset, 10,000 BC, i.e., for 12 millennia.

In Fig. 2, for the Roman Empire times, the global population change $\Delta P\approx 0$, and then ${G}_{P}\approx 0$ (see the definition of RGR given above). It leads to an anomaly in the scale ${{log}_{10}G}_{P}$ used in Fig. 2. After the fall of the Empire, particularly from 700 to 800 CE, the general trend of strong ${G}_{P}\left(t\right)$ increase appears. It lasts till the present, with a single strong disturbance related to the Black Death times. Figure 2, and its extension in Supplementary Information also show significant historical periods and dates correlated with emerging hallmarks detected due to the derivative analysis detecting subtle distortions. Particularly notable is the emergence of domains associated with the compressed ($\beta >1$) and stretched ($\beta <1$) exponential behavior in SM-1 Eq. (3). The most evident is the crossover occurring near the year 1970. It led to the stretched exponential behavior that has continued till nowadays. Such behavior indicates the slowing down rate of the population growth. Notwithstanding, it is related to the rise of the global population from ca. 3 billion to 8 billion in the last 5 decades. Interestingly, such 'crossovers’ also appear for earlier periods, as visible in Fig. 2. Perhaps the most interesting is the crossover related to the year ~ 1750. This is when the first achievements of the 1st Industrial Revolution were implemented and developed. It was also the beginning of significant social and political changes and great wars. From the year ~ 1815, correlating with the Vienna Congress establishing a new 'order' in nineteenth century Europe, the period of the global population's 'compressed' rise $(\beta >1)$, continued till the year 1970 takes place.

The plot covering the domain from 10,000 BC, i.e., the Holocene onset, till 2023 is presented in Supplementary Information, Fig. S1. It shows that the population changes in prehistoric times can also be SM-1 type, with the mentioned crossover from the early Neolithic to the late Neolithic epoch. A manifestation of the subsequent Bronze Age epoch, the times of the first grand civilizations and urban centers formation^98^, is also visible.

Table 1 summarizes results obtained via the distortions-sensitive analysis related to Eqs. (3) and (7) and Fig. 2 and Fig. S1. Periods given in Table 1 are slightly broader because they are related to periods emerging after the portrayal using SM-1 Eq. (3) with parameters derived from the analysis presented in Fig. 2.

**Table 1:** Values of relevant parameter related to Super-Malthus, empowered exponential, Eq. (3) for subsequent domains of it possible validated application revealed via the distortions-sensitive analysis defined by Eq. (7), which results applied for tested global population data are shown in Fig. 2 and Fig. S1.

Period (years)	Population change (millions)	Exponent $\beta$	Relaxation time $\tau$ (year)
$1970 \to 2023$	$3000 \to 8000$	$0.85\pm 0.03$	$72.7\pm 2$
$1929 \to 1978$	$2061 \to 2488$	$1.52\pm 0.06$	$58.6\pm 2$
$1790 \to 1948$	$920\to 2488$	$1.4\pm 0.1$	$160\pm 4$
$1690 \to 1760$	$580 \to 745$	1.2 ± 0.2	$150\pm 20$
$1520\to 1700$	$455 \to 590$	0.8 ± 0.1	$150\pm 10$
$1430 \to 1520$	$360 \to 455$	‘Return’ after the Black Death	
$1355 \to 1430$	$455 \to 360$	The Black Death decay	
$500 \to 1350$	*A reliable fit in domains indicated in *Fig. 2* is not possible (too small number of scattered data)*		
$30 BC \to 500 AD$ Roman Empire	~ (190–200)	$\beta =0 or \tau =\infty$ in SM-1 Eq. (3)	
$400 \to 200$ (BC)	~ (140–148)	*Anomalous behavior, close to Roman Empire times (above)*	
$3000 \to 1000$ (BC) Bronze Age	$\sim (15\to 50)$	$\beta \approx 1$	$900$
$4800 \to 3000$ (BC) Late Neolithic & early Bronze Age	$\sim (4 \to 15)$	$\beta \sim 1.2$	$1500$
$\mathrm{10,000} \to 4800$ (BC) Early Neolithic	$\sim (1 \to 4)$	$\beta \approx 1$	$8900$

The inset in Fig. 2 focuses on the Industrial Revolutions times. The approximation by two linear domains with a positive sign of the slope, related to the exponent $\beta >1$, is possible from 1815 to about 1948 and then from 1948 to 1970. Notable is the coincidence with the end of the basic recovery after World War 2 (1948), (iii) the onset of the grand economy crisis (Black Friday: 24th Oct., 1929). historical data, namely: (i) the post-Napoleonian congress in Vienna, which established the new order, lasting at least for the nineteenth century, (ii) after the year ~ 1970 the transition to the behavior associated with the unique in the global population growth history exponent $\beta <1$, lasting till nowadays, takes place. However, this does not mean a decline in the global population; it only means weaker relative growth compared to previous periods. Maintaining such a trend, based on SM-1 Eq. (4) with the exponent $\beta = 0.85$ and $\tau =72.1$ (year) suggests the global population of $8.971 billion$ in 2030, $11.341 billion$ in 2050 and $19.98 billion$ in 2100.

**Figure 3:**
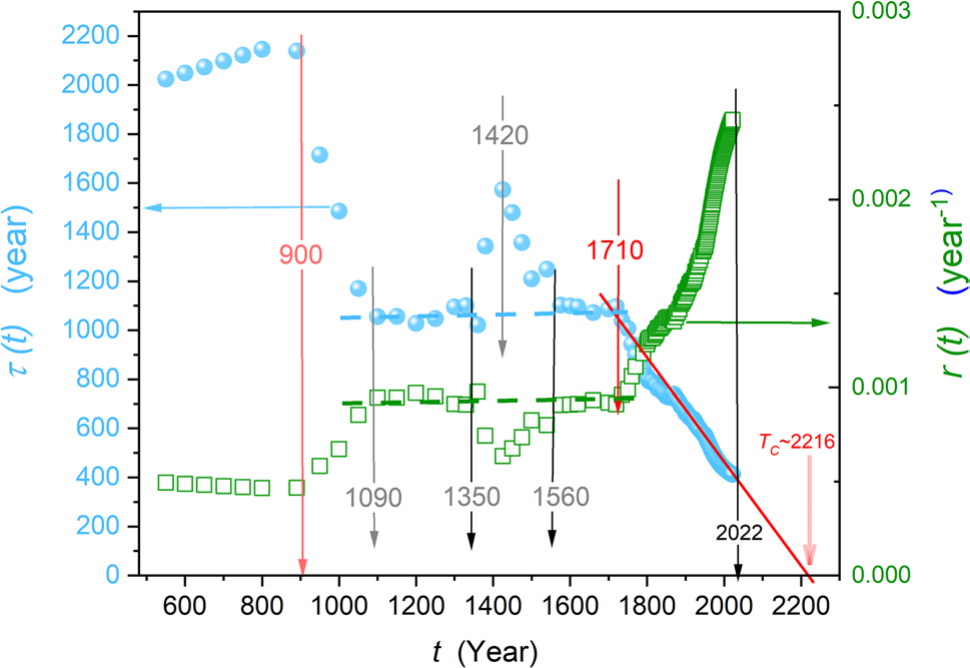
The evolution of relaxation time *τ*$\left(t\right)$ (full circles, light blue & the left scale) and the rate $r\left(t\right)$ (squares, green & the right scale) coefficients, recalling the S-M-2 Eq. (4) and calculated via Eq. (8). Vertical arrows indicate emerging characteristic times. Dashed horizontal lines are for the basic Malthus behavior with *τ* & $r=const$.

Figure 3 is related to the SM-2 Eq. (4). As mentioned above, its direct application for describing population data is puzzling. Notwithstanding, it can serve as the base for testing the time evolution of the relaxation time $\tau \left(t\right)$ or alternatively, the apparent growth rate coefficient $r\left(t\right)=1/\tau \left(t\right)$, as shown in Eq. (8). The plot is related to the period after the fall of the Roman Empire, i.e., the onset of Medieval times. From 500 to 900 CE, there is a slight linear increase in the relaxation time (decrease in the growth rate). Between years ~ 900 and ~ 1100, there is a 50% decrease of $\tau \left(t\right)$ which for SM-2 Eq. (4) means speeding up population growth. Surprisingly (for the authors), from 1100 to 1700, the dashed horizontal linear behavior appears. It indicates the averaged trend described by $\tau \left(t\right)=const$ or alternatively $r\left(t\right)=const$, related to the basic Malthus dependence (Eq. 1). It is distorted by the impact of the most terrible world pandemic, the *Black Death*, whose influence is detectable for 200 years. From the eighteenth-century onset, the evolution of the relaxation time can be approximated by a linear function, shown by the red line. Consequently, the parameter $r\left(t\right)$ increases following the hyperbolic function, also visible in Fig. 3.

The year ~ 1700 is the onset of the Enlightenment Age, lasting till the end of the eighteenth century, and also the Industrial Revolutions times, lasting till today. In this period, the linear decrease in relaxation time takes place: $\tau \left(t\right)=a-bt$, leading to the following specification of SM-2 Eq. (4):

9 $$P\left(t\right)={P}_{0}exp\left(\frac{t}{\tau \left(t\right)}\right)={P}_{0}exp\left(\frac{t}{a-bt}\right)={P}_{0}exp\left(\frac{t/b}{a/b-t}\right)={P}_{0}exp\left(\frac{b{\prime}\times t}{{T}_{c}-t}\right)$$

where $t=0$ is for the 1720 onset year and ${P}_{0}\approx 610 million$—the estimated population for this year; ${b}{\prime}=1/b$ and the ‘critical time’ ${T}_{C}=a/b$.

The form of the right-hand part of Eq. (9) is worth stressing. It resembles the critical-like behavior in constrained system complex systems, for instance, in critical phenomena physics within the so-called Griffiths model^107–109^. For the parameters that can be derived from the results presented in Fig. 3, the following relation approximates global population changes in the Industrial Revolutions period:

10 $$P\left(t\right)={P}_{0}exp\left(\frac{b{\prime}\times t}{{T}_{c}-t}\right)=610\times exp\left(\frac{1.62t}{2216-t}\right)$$

The above equation and Fig. 3 shows the ‘critical time’ ${T}_{C}\approx 2216\mp 3$ for which $\tau \left(t\to {T}_{C}\right)\to 0$ or alternatively $r\left(t\to {T}_{C}\right)\to \infty$, can mean an infinite world population, i.e., the Doomsday or ‘Dooms-year’. Nevertheless, one should not expect such a catastrophe. In general, for complex systems under constraint, one can expect a relatively continuous passage (‘tunneling’) through the catastrophic singularity rather than infinite values associated with the unconstrained singularity for $T\to {T}_{C}$).

## Conclusions

The report discusses the evolution of the global population from the Holocene onset till 2023. It is based on an extensive data set collected from various sources and subjected to a numerical filtering procedure to obtain the optimal data set. The global population is considered within extensions of the reference Malthusian Eq. (1): (1) the empowered Malthusian equation, referring to the KWW and Weibull dependences, (2) the Malthus-type equation with time-dependent growth rate $r\left(t\right)$, or alternatively, relaxation time $\tau \left(t\right)$. They are named Super Malthus equations and indicated as SM-1 and SM-2.

The analysis starts with distortions-sensitive tests of empirical data, for which linear domains indicate regions of their validated applicability of the Super Malthus description. Finally, the obtained scaling equation can be applied to these regions using determined optimal parameter values. It is the ‘bottom-up’ approach. Usually, models related to scaling equations are used for an arbitrarily selected time domain. One can call it the ‘up-down’ approach.

The distortions-sensitive analysis carried out in frames of Super Malthus relations, namely SM-1 (Eq. 3) and SM-2 (Eq. 4) showed that the pattern of global population growth is non-monotonic. Significant crossovers are associated with the crossover between Neolithic and Paleolithic times, the fall of the Roman Empire, the year ~ 1100, and the onset of Industrial Revolutions. Approximately linear changes of $\tau \left(t\right)=a-bt$, lead to the critical, constrained Eq. (10) with the ‘critical’ Doomsday/Dooms-year at the year ${T}_{C}\approx 2026$, explicitly manifested in Fig. 3. Such behavior has to recall the most famous Doomsday scaling equation by von Foerster et al.^110^ published in 1960:

11 $$P\left(t\right)=\frac{A}{{\left(D-t\right)}^{\gamma =0.99}} \Rightarrow P\left(t\right)=\frac{A}{D-t}$$

where $D=2026.9$ is for the ‘Doomsday”, with the infinite global population.

The relation was proven by the ${log}_{10}P\left(t\right)$ vs. ${log}_{10}t$ with the time reference to the fall of the Roman Empire. It uses 26 population data from 400 CE to 1958, showing excellent scaling quality. In Ref.^110^ the exponent $\gamma =0.99$ was declared, but taking into account the realistic fitting error ± 0.03, one obtains the hyperbolic equation shown on the right side of Eq. (11). The Doomsday or hyperbolic Eq. (11) met with extraordinary interest and strong criticism because of the spectacular prediction^111–121^. However, starting in the 1970s and 1980s, increasing deviations from Eq. (11) were observed, fortunately showing that von Forster Doomsday would not occur. Also, the emergence of numerous new data for the global population has shown that the quality of the fit may not be as perfect^69^ as suggested in Ref.^110^.

It is worth noting that the decomposition of the ‘critical-like’ SM-2 Eq. (9) in a Taylor series reproduces hyperbolic von Forester Doomsday Eq. (11):

12 $$P\left(t\right)={P}_{0}exp\left(\frac{b{\prime}\times t}{{T}_{c}-t}\right) \Rightarrow P\left(t\right)={P}_{0}\left(1+\frac{b{\prime}\times t}{{T}_{C}-t}+\dots \right)\propto \frac{B}{D-t}$$

The estimate of the critical time (i.e., 'Doomsday’) from the right side of Eq. (12) must be significantly smaller than after expansion on the left, because subsequent expansion terms are omitted, i.e.: ${T}_{C}\gg {T}_{C}{\prime}$.

Recalling studies of critical behavior in physical complex systems under constraints, one can expect that the critical behavior associated with 'Doomsday' appears only remote from the critical singularity at ${T}_{C}$. For $T\to ({T}_{C},D)$ one should expect the crossover to the finite value behavior, i.e., avoiding criticality (‘Doomsday’) behavior. The year of ${T}_{C}$ should be passed without the Doomsday singularity.

Results presented above also show one more unique for the global population growth occurring in the last decade, namely the crossover for the exponent *β* > 1 for (*t* < 1970) → *β *< 1 for (t > 1970). Recalling references to physical models above, the exponent $\beta >1$ is related to the ‘fastened’ development, associated with the appearance of new ‘ordered nucleation centers’ and ‘internal energy’ creation. The case $\beta <1$ is related to energy dissipation and a rather disordered system. Is it also the case of global population development? The question also arises as to why the crossover occurred near 1970. A lot happened then. It was a kind of cultural revolution in many countries, the definitive end of the era of colonization, and soon an oil crisis. However, according to the authors, it was then that significant implementations of the new generation of electronics appeared, which in the next decade began the digital era of computers and accelerating information exchange. For the economy, this is a time of a noticeable shift towards services, especially financial ones.

This report demonstrates the possibilities of describing global population changes using Super Malthusian equations, allowing for complementary interpretations based on results obtained in studies of complex systems and soft matter in physics. Notable that such a parallel indicates the spontaneous creation of ‘aggregation centers’ characterized by differences in local dimensionality, as shown in the discussion of the KWW relation or the Avrami model above. For the global human population, it can mean that the emergence of first cities or civilizations can be the inherent feature of the complex system development with some ability to interact. The authors also indicate the discussion of the empowered exponential SM-1 (Eq. 3) behavior within KWW, Weibull, and Avrami models, which offers messages related to the distribution of relaxation times, coupled to underlying dynamic processes hidden in different values of the $\beta$ power exponent, determined from testing time-related population trends. This report is related to the global population changes, but the link to the mentioned models, data preparation route, and the distortions-sensitive analysis can be used to study any population dynamics. This work also indicates that global population changes may be non-monotonic, posing a significant problem for long-period deterministic forecasting. Essential is introducing the distortions-sensitive data analysis, leading to the bottom-up approach implemented in the report.

## Methods

The results presented in this report explore a new way of preparing global population data based on collecting data from different sources and their numerical filtering using the protocol introduced by one of the authors (ADR) in materials engineering and glass transition physics studies^58–66^. The optimal time-related evolution path in a set of scattered data is determined using the Savitzky-Golay^67^ principle protocol with the support of Origin and Mathematica software. In the first step, 'outlying population data' that deviate from the general time trend are removed. They are related to data that differ by more than $3x\sigma$ (3 standard deviations) from the general trend. In the next step, numerical filtering, focused on the impacts of successively changed levels, was performed. This report examines global population data from Refs.^68–75^. As a result of the mentioned protocol, a 'smooth' set of 197 population data from 10,000 BC to 2023 has been determined. These data allowed for the derivative analysis, avoiding problems with ‘native’ global population data that can simultaneously differ even by 100%. The derivative-based and distortions-sensitive analysis described below can reveal time domains in which given model equations, namely SM-1 Eq. (3) and SM-2 Eq. (4), can be used for describing population data. Related dependencies are presented in the next section. This procedure also yields optimal values of parameters with well-defined uncertainties/errors and reduces the final fitting of $P\left(t\right)$ data solely to prefactors ${P}_{0}$ in Eqs. (3) and (4). The authors want to indicate that estimates of the global population, as we move away from modern times, undergo significant corrections related to new results and multidimensional research results. One example is the pre-Columbian population in South America, which appears to be qualitatively more significant than was thought a decade ago^122,123^. This indicates that the analysis of global population trends must be continued, with a critical discussion of available empirical data.

### Supplementary Information

Supplementary Information.

## Data Availability

The datasets used & analyzed in presented studies are available from the corresponding author upon reasonable request.
