# Supplementary material for: Global population: from Super-Malthus behavior to Doomsday criticality
Source: Sci Rep. 2024 Apr 29;14:9853. doi: 10.1038/s41598-024-60589-3 (PMC11058850; doi:10.1038/s41598-024-60589-3)
Supplement: Supplementary file 1 — Supplementary Information. [file 41598_2024_60589_MOESM1_ESM.pdf]

Supplementary Information

The report considers the portrayal of the global population evolution $P(t)$ via the 'empowered' exponential function, called the 'Super Maltus' SM-1 equation. It is given by Eq. (3) in the main body of the report, namely:

$$P(t) = P_0 exp\left[\pm\left(\frac{t}{\tau}\right)\right]^{\beta} \quad \Rightarrow \quad lnP(t) = lnP_0 \pm (t/\tau)^{\beta} \qquad \text{(S1)}$$

where τ denotes the relaxation time, related to the apparent growth rate coefficient:

$r(t) = 1/\tau(t)$.

For the basic Malthus dependence (Eq. (1) in the main body of the report) the exponent $\beta = 1$ and $r(t) = r = const.$

Subsequently, the distortions-sensitive and derivative-based analysis focused on finding time domains in which the validated application of the above relation is given by Eq. (7) in the main body of the report, namely:

$$y(t) = log_{10}\left[\frac{dlnP(t)}{dt}\right] = log_{10}G_P(t) = log_{10}\left(\frac{\beta}{\tau^{\beta}}\right) + (\beta - 1)log_{10}t = A + B \times x \qquad \text{(S2)}$$

$G_P(t)$ denotes the per capita relative growth rate (RGR), recalling its analytic formulation presented in the given report.

Figure 2 in the main body of the report shows the analysis results via the above relation covering the 2 last millennia, enabling the detailed insight.

Figure S1 below presents the results of such distortions-sensitive analysis for the whole time domain considered in the report, from the Holocene onset till 2023, i.e., covering **12 millennia**.

Table I in the main body of the report summarizes the results presented in Figure 1 and Figure 1, also giving values of relevant parameters.

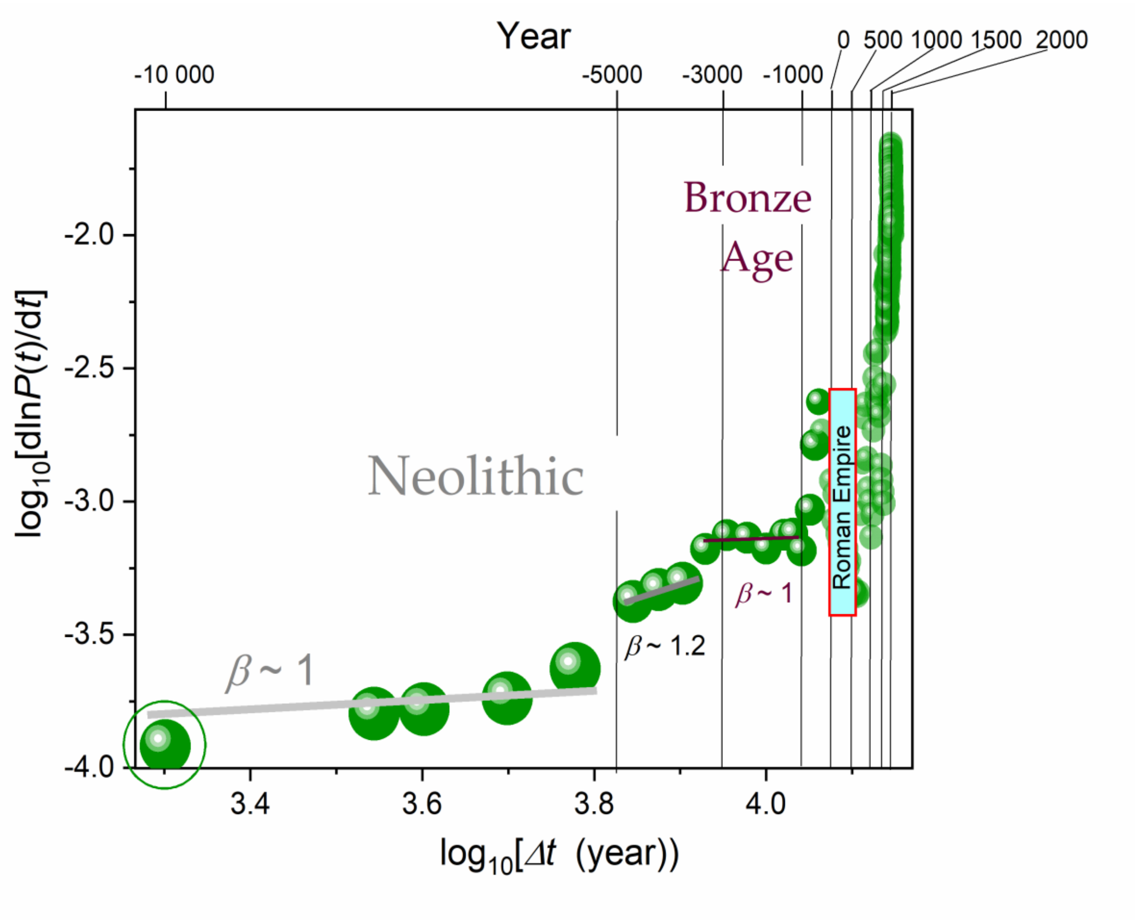

Figure S1 The derivative based-plot related to presenting the evolution of per capita relative growth rate (RGR) in the log-log scale, taking $t_{ref.} = 12\ 000\ BC$ as the reference onset. It supplements the results presented in Figure 2 in the main body of the report, focused on the period from the Roman Empire times to the present ones. The presentation recalls Eq. (7) in the report and Eq. (S2) above. Linear domains in Fig. S1 and Fig. 2 indicate domains where the empowered exponential description, namely SM-1 Eq. (3), recalled above as Eq. (S1), can be applied. The changing size of the circles indicates the uncertainty. The plot applies the log-log scale, in agreement with Eq. (S1), which also facilitates the insight into the presentation covering the long period of 12 millennia. The upper scale shows directly BC ('-') and AD ('+') times.
